# Behavior of Phosphorus During Selective Reduction of Iron from Oolitic Ore and Separation of Reduction Products

**DOI:** 10.3390/ma18174051

**Published:** 2025-08-29

**Authors:** Bakyt Suleimen, Almas Yerzhanov, Nurlybai Kosdauletov, Galymzhan Adilov, Assylbek Nurumgaliyev, Assemay Pushanova, Bauyrzhan Kelamanov, Pavel Gamov, Konstantin Smirnov, Talgat Zhuniskaliyev, Yerbol Kuatbay, Assylbek Abdirashit

**Affiliations:** 1 Department of Metallurgy and Materials Science, Karaganda Industrial University, Temirtau 101400, Kazakhstan; suleimenb@susu.ru (B.S.); adilovg@susu.ru (G.A.); a.nurumgaliyev@tttu.edu.kz (A.N.); a.pushanova@tttu.edu.kz (A.P.); ye.kuatbay@tttu.edu.kz (Y.K.); 2 Department of Pyrometallurgical and Foundry Technologies, South Ural State University, Lenin Prospekt 76, Chelyabinsk 454080, Russia; gamovpa@susu.ru (P.G.); smirnovk@susu.ru (K.S.); 3 Department of Metallurgy and Mining, K. Zhubanov Aktobe Regional University, Aktobe 030000, Kazakhstan; bkelamanov@zhubanov.edu.kz; 4 Department of Science, Eurasian Technological University, Almaty 050000, Kazakhstan; t.zhuniskaliyev@etu.edu.kz

**Keywords:** oolitic iron ore, phosphorus, hydrogen reduction, smelting, metallic iron, phase separation, magnetic separation, metal and slag

## Abstract

This study investigates the behavior of phosphorus during high-temperature smelting of hydrogen-reduced high-phosphorus oolitic iron ore from the Lisakovsk deposit. The preliminary reduction was carried out at temperatures ranging from 600 to 900 °C using hydrogen, aiming to selectively reduce iron to the metallic phase while retaining phosphorus in the oxide form. The resulting reduced products were subjected to wet magnetic separation and liquid-phase separation. It was found that neither method provides effective separation of phosphorus from iron: phosphorus partially enters the magnetic fraction and, during smelting, transfers into the metallic phase. To confirm the mechanism of phosphate reduction by metallic iron, a control experiment was conducted, in which a mixture of reduced iron and raw ore was smelted at 1650 °C. Microstructural and elemental analyses confirmed the redistribution of phosphorus into the metallic phase. These findings indicate that effective separation of iron and phosphorus cannot be achieved by reduction roasting alone and highlight the need for further studies on slag formation conditions and phase separation kinetics.

## 1. Introduction

The increasing phosphorus content in iron ore raw materials remains a serious challenge for the metallurgical industry, as even trace amounts significantly degrade the mechanical properties of steel. Oolitic iron ores, in particular, are especially difficult to process. These ores are widely distributed in France, Germany, the USA, Canada, Pakistan, China, Kazakhstan, and other countries [1,2,3]. Due to their fine-grained structure, such ores are not amenable to conventional beneficiation methods, and effective phosphorus removal is only possible during the metallurgical processing stage [4,5,6,7,8,9].

Various approaches to dephosphorization include hydrometallurgical, pyrometallurgical, physicomechanical, and biochemical methods. Hydrometallurgical techniques—such as acid leaching with H_2_SO_4_ or HCl—can remove up to 90% of phosphorus but are associated with considerable iron losses, high reagent consumption, and environmental concerns [10,11,12]. Pyrometallurgical methods—involving reduction with carbon or carbon monoxide, followed by smelting—can reduce phosphorus content to around 0.3%, but they require high temperatures and the use of dephosphorizing agents [13,14,15]. Magnetic separation and flotation are effective only when phosphorus is present in discrete phosphate minerals; however, in the case of uniformly distributed phosphorus—as in oolitic ores—these methods prove largely ineffective [16,17,18]. Biochemical methods demonstrate selectivity but suffer from extremely slow reaction rates and are not applicable on an industrial scale [19,20,21].

Thus, existing methods for phosphorus removal from iron ores have significant limitations and fail to solve the problem of dephosphorization for complex types such as high-phosphorus oolitic ores.

One of the promising approaches is selective solid-state reduction of iron [22,23,24]. This method is used not only for iron ores but also for chromite, manganese, titanomagnetite ores, and various types of industrial waste [25,26,27,28,29].

Our previous studies have shown that, in oolitic ore from the Lisakovsk deposit, it is possible to selectively reduce iron using carbon monoxide or hydrogen while retaining phosphorus in the oxide phase [30].

In this study, an attempt was made to separate the reduced iron from phosphorus-containing slag phases using two methods:

- wet magnetic separation;
- liquid-phase separation during smelting.

Both approaches aimed to obtain an iron phase purified from phosphorus. However, in both cases, it was found that phosphorus partially enters the metallic phase, despite being initially present in oxide form after reduction roasting. This effect was particularly pronounced during high-temperature smelting, where the formation of phosphides and the incorporation of phosphorus into the metal structure were observed. These findings contradict the expected outcome and call into question the effectiveness of the proposed separation scheme.

To test the hypothesis that phosphorus can be reduced by metallic iron during smelting, a control experiment was conducted. A mixture of reduced iron and raw ore (at a 1:1 ratio) was subjected to high-temperature treatment at 1650 °C in a vacuum furnace. The resulting products were analyzed using scanning electron microscopy (SEM) and energy-dispersive X-ray spectroscopy (EDS), which allowed us to track the redistribution of phosphorus and confirm its reduction and subsequent transfer into the metallic phase.

Unlike most previously published studies focused on thermodynamic calculations or liquid-phase metal–slag separation, the present work examines the behavior of phosphorus during the processing of high-phosphorus oolitic iron ores, taking into account the mechanism described by the electronic theory of reduction. This approach provides a new perspective on the processes of phosphorus redistribution and helps identify directions for improving its separation efficiency. The results have practical significance for the processing of ores from the Lisakovsk and Ayatsk deposits, which together account for more than 60% of Kazakhstan’s iron ore reserves.

The objectives of this study are to evaluate the effectiveness of separating reduced products of high-phosphorus oolitic ore after hydrogen reduction, to identify the causes of phosphorus transfer into the metallic phase during smelting, and to experimentally confirm the possibility of phosphorus reduction by metallic iron.

## 2. Materials and Methods

The starting material was oolitic iron ore from the Lisakovsk deposit; its chemical and phase composition have been reported in previous studies [30].

### 2.1. Hydrogen Reduction

Samples of high-phosphorus oolitic iron ore (~1 mm, ~50 g) were subjected to hydrogen reduction in an RB Automazione MM 600 (Genoa, Italy) laboratory tube furnace at four different temperatures: 600, 700, 800, and 900 °C. The selected temperature range was based on the thermodynamic characteristics of iron reduction by hydrogen. According to the Ellingham diagram [31], the reduction of iron oxides with hydrogen becomes thermodynamically feasible and kinetically effective starting from 600 °C, whereas the reduction of phosphorus compounds requires significantly higher temperatures (above 1000 °C). This enables selective reduction of iron while retaining phosphorus in the oxide phase.

At each temperature, separate experiments were carried out with a fixed holding time and gas flow rate. High-purity hydrogen (99.99% H_2_) was used as the reducing agent and supplied at a flow rate of 1 L/min. This flow rate ensured sufficient reduction kinetics, maintenance of a reducing atmosphere, effective removal of water vapor, and prevention of iron reoxidation by secondary reaction products. Prior to the introduction of hydrogen, the reaction chamber was purged with argon at a flow rate of 0.5 L/min. Argon purging was performed continuously from the beginning of heating until the target temperature was reached, in order to eliminate oxygen and prevent oxidation of iron in the early stage of the experiment.

Samples were placed in an open mesh container made of heat-resistant steel to ensure free circulation of hydrogen within the reaction zone. This design minimized the accumulation of reduction gases and improved the reaction conditions. Upon reaching the target temperature, hydrogen flow was initiated, and the isothermal holding period began. The duration of isothermal reduction was 60 min. After the experiment, the furnace was shut down, and cooling was carried out under an argon atmosphere.

The reduced products were extracted and partially analyzed using X-ray diffraction (XRD) on a Rigaku Ultima IV diffractometer (Rigaku, Tokyo, Japan) and scanning electron microscopy (SEM) on a JEOL JSM-7001F microscope (JEOL, Tokyo, Japan) equipped with an OXFORD X-Max 80 energy-dispersive X-ray spectrometer (EDS) (Oxford Instruments, Abingdon, UK) for elemental analysis. The quantitative evaluation of the phase composition of the reduced samples was carried out using Match! 3 (Crystal Impact, Bonn, Germany) software based on the XRD data. The remaining portion of the samples was used for separation experiments, which involved two approaches: magnetic separation, and liquid-phase separation.

### 2.2. Hydrogen Reduction

To separate the phases, the products of reduction roasting were subjected to wet magnetic separation. The samples were first manually ground in an agate mortar to a particle size of less than 0.1 mm. The separation was carried out using a laboratory CXG-ZN50 magnetic separator (Ganzhou, Jiangxi Province, China) under the following conditions: magnetic field strength of 250 mT, feed rate of 40 mm/s, magnetic drum diameter of 27 mm, and a working zone diameter of 25 cm. The mass of each sample was approximately 20 g.

Separation was performed in a single-pass mode for samples reduced at temperatures of 600, 700, 800, and 900 °C. The obtained magnetic and non-magnetic fractions were collected, dried, and weighed. In all cases, the non-magnetic fraction contained a fine-dispersed component that was partially lost with the water used for equipment cleaning. These residues were difficult to filter, resulting in minor mass losses.

All collected fractions (magnetic and non-magnetic) were subjected to X-ray diffraction (XRD) analysis to determine their phase composition.

### 2.3. Liquid-Phase Separation

**Figure 1:**
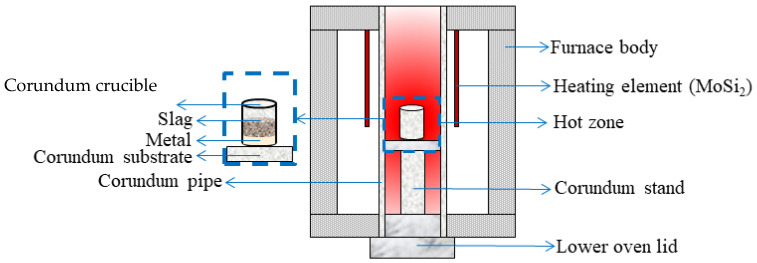
Schematic diagram of the experiment for smelting hydrogen-reduced oolitic ore samples in a vertical resistance furnace.

Liquid-phase separation was carried out using an open vertical alumina tube resistance furnace (Nabertherm, RHTV 120–300/18, Lilienthal, Germany) equipped with four molybdenum disilicide heating elements and a type B platinum–rhodium thermocouple. The experimental setup is illustrated in Figure 1.

A corundum crucible containing the magnetic fraction of hydrogen-reduced oolitic ore (reduced at 600–900 °C and separated by wet magnetic separation) was placed into a preheated resistance furnace at 1650 °C. The material was heated to the melting temperature and held for 1 min. After this holding period, the crucible was removed and cooled to room temperature. The resulting metal and slag samples were analyzed using electron microscopy combined with micro-X-ray spectroscopy to determine their elemental composition. The analysis allowed for the identification of the phase composition and the distribution of elements in the separated products.

In addition, to verify the hypothesis that phosphorus can be reduced by metallic iron during high-temperature smelting, a control experiment was conducted under vacuum conditions. Pure metallic iron (previously obtained by hydrogen reduction) was mixed with the original oolitic ore at a 1:1 mass ratio. The mixture was placed into a corundum crucible and loaded into a high-temperature vacuum furnace (Nabertherm VHT 100/22-GR, Lilienthal, Germany).

The furnace was heated to 1650 °C, and the sample was held isothermally for 30 min under constant vacuum. After smelting, the crucible was cooled to room temperature inside the furnace. The resulting products were extracted and separated into metallic and slag phases for further analysis using scanning electron microscopy (SEM) and energy-dispersive X-ray spectroscopy (EDS).

## 3. Results and Discussion

### 3.1. Hydrogen Reduction

**Figure 2:**
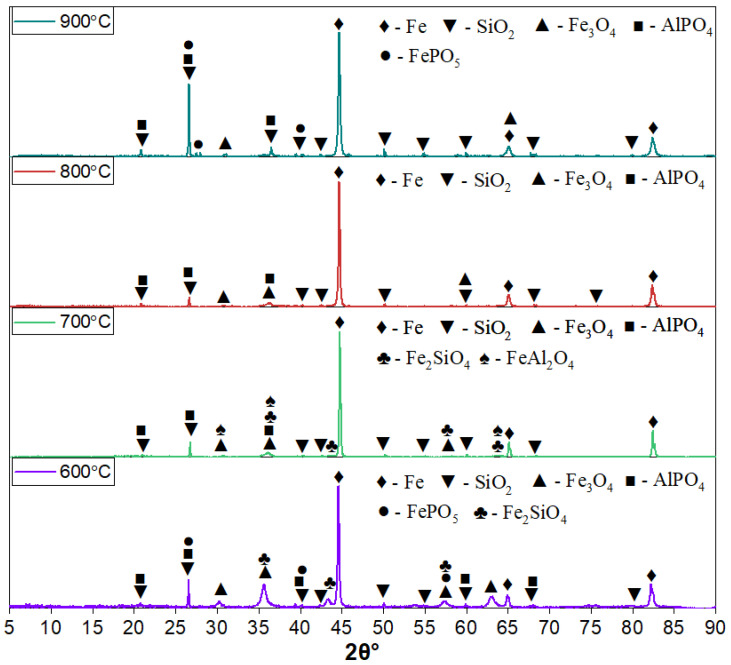
XRD patterns of hydrogen-reduced oolitic ore samples at 600, 700, 800, and 900 °C. The holding time was 60 min, and the hydrogen flow rate was 1 L/min.

Figure 2 presents the XRD patterns of oolitic ore samples reduced by hydrogen at temperatures of 600, 700, 800, and 900 °C (holding time—60 min, H_2_ flow rate—1 L/min).

All samples exhibit intense diffraction peaks of metallic iron (α-Fe), which are most prominent at 800 and 900 °C, indicating a high degree of reduction. Magnetite (Fe_3_O_4_) peaks are still present, particularly at 600 and 700 °C, along with residual phases such as quartz (SiO_2_) and aluminum phosphate (AlPO_4_). At 600 °C, distinct fayalite peaks are observed. As the temperature increases to 700 °C, the intensity of the fayalite peaks decreases and hercynite peaks appear. With further temperature increases to 800 and 900 °C, both fayalite and hercynite reflections nearly disappear.

The iron phosphate phase (FePO_5_) is detected in samples reduced at 600 and 900 °C. At 700 and 800 °C, FePO_5_ reflections are either absent or significantly suppressed due to the dominance of more intense peaks from other phases such as Fe, SiO_2_, Fe_3_O_4_, and AlPO_4_.

The results of the quantitative X-ray phase analysis of the reduced samples are shown in Figure 2. With increasing reduction temperature, a consistent increase in the metallic iron (Fe) content is observed, accompanied by a corresponding decrease in oxide phases.

**Figure 3:**
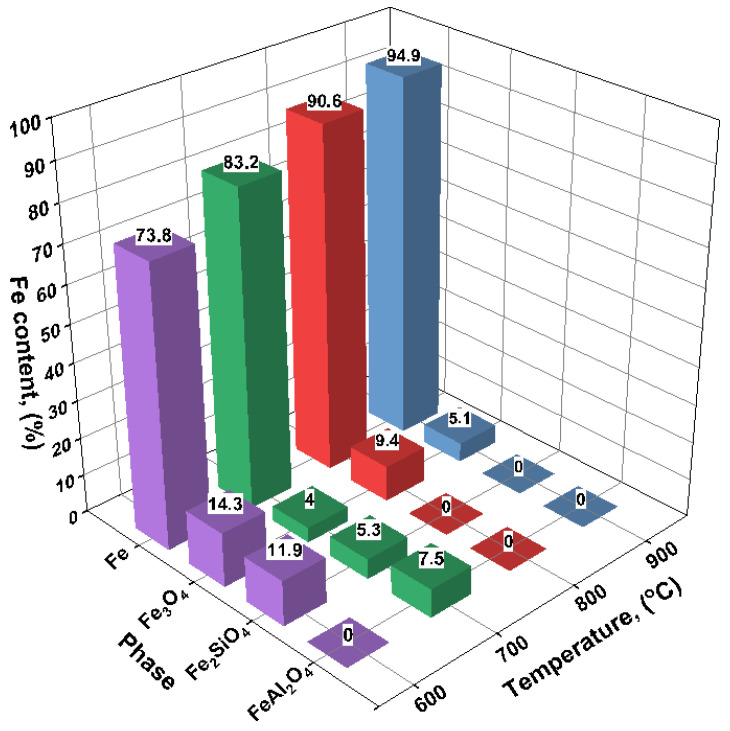
Content of iron-containing phases after hydrogen reduction roasting of oolitic ore samples at 600, 700, 800, and 900 °C.

At 600 °C, the product predominantly consists of metallic iron (73.8%), although a significant amount of magnetite (Fe_3_O_4_—14.3%) remains. In addition, a silicate phase—fayalite (Fe_2_SiO_4_, 11.9%)—is detected, which likely forms through the interaction of FeO with silicon dioxide under incomplete reduction conditions (Figure 3).

At 700 °C, the reduction becomes more complete: the Fe content increases to 83.2%, while the amount of Fe_3_O_4_ decreases to 4.0%. Fayalite (5.3%) and hercynite (FeAl_2_O_4_, 7.5%) are still present in the phase composition, indicating reactions between iron oxides and the aluminosilicate matrix of the ore.

At 800 °C, the degree of reduction reaches 90.6%, with oxide phases nearly eliminated: only 9.4% magnetite is detected, and silicate or aluminate phases are no longer observed.

At 900 °C, the metallic iron content reaches a maximum of 94.9%, and the magnetite content drops to 5.1%. No silicate or aluminate phases are detected, suggesting effective reduction and the absence of notable reactions between iron and gangue components.

Thus, as the reduction temperature increases from 600 to 900 °C, a gradual decrease in oxide phases and an accumulation of metallic iron are observed. The presence of fayalite and spinels at 600–700 °C may be attributed to residual FeO reacting with SiO_2_ and Al_2_O_3_ from the gangue matrix. The complete disappearance of these phases at 800–900 °C indicates full reduction and thermodynamic stabilization of α-Fe.

**Figure 4:**
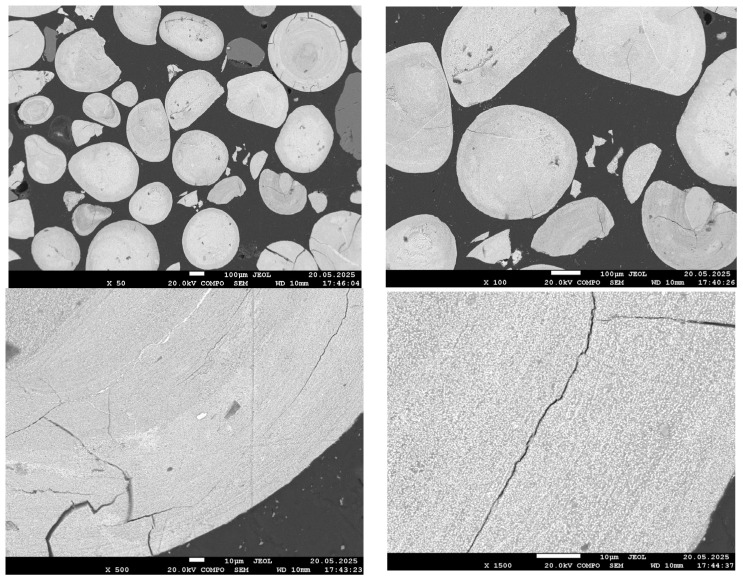
SEM images of the reduced sample at 600 °C.

**Figure 5:**
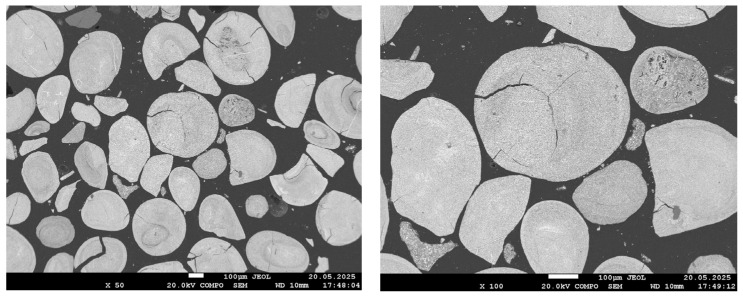

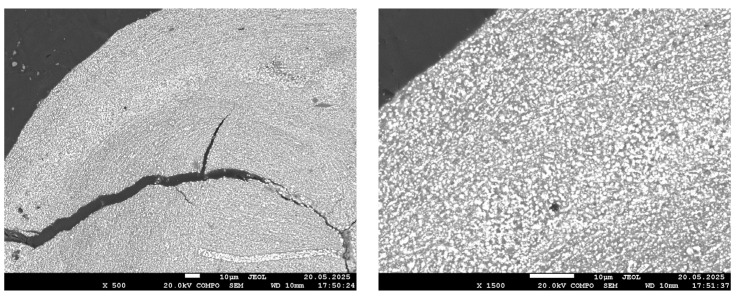
SEM images of the reduced sample at 700 °C.

**Figure 6:**
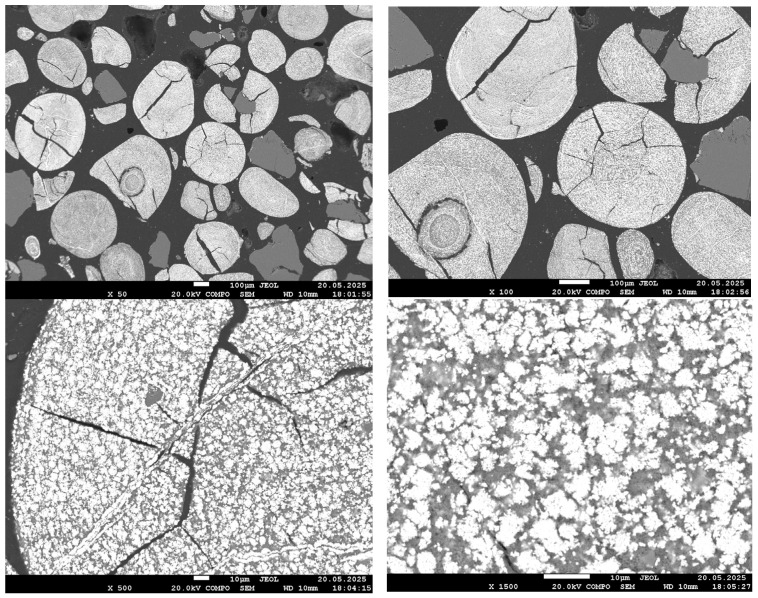
SEM images of the reduced sample at 800 °C.

**Figure 7:**
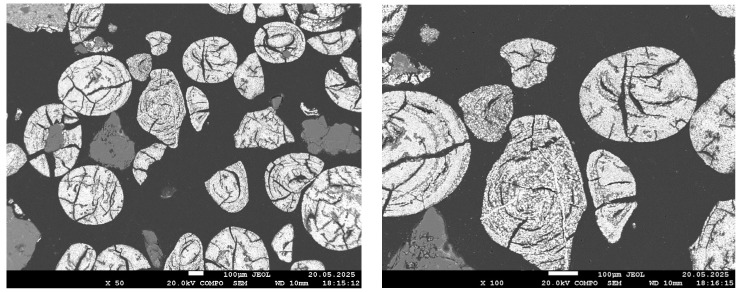

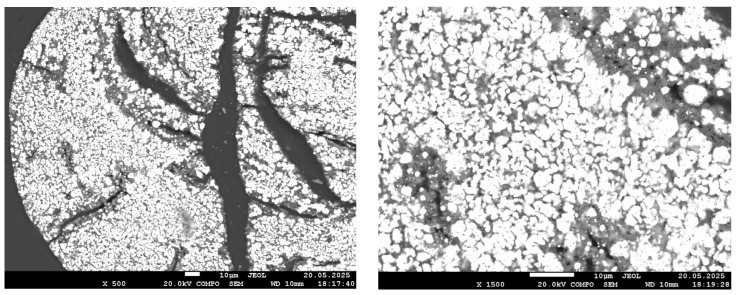
SEM images of the reduced sample at 900 °C.

Figure 4, Figure 5, Figure 6 and Figure 7 present SEM images of hydrogen-reduced oolitic iron ore samples at temperatures of 600, 700, 800, and 900 °C, respectively. Morphological studies were conducted at various magnifications—from general structure overview (×50–×100) to local microzone analysis (×500–×1500). Bright regions in the images correspond mainly to areas enriched in metallic iron, while dark regions represent oxide and silicate phases.

The morphology of the reduced sample at 600 °C is nearly identical to that of the original oolitic ore. In the low-magnification images (×50–×500), bright areas corresponding to metallic iron are virtually absent. In certain regions, small cracks are observed, likely associated with the decomposition of goethite and structural stresses during heating. At higher magnification (up to ×1500), faint bright zones become barely distinguishable; these may correspond to initial regions of iron reduction.

At 700 °C, the sample morphology at low magnifications (×50 and ×100) appears similar to that at 600 °C: an oxide surface remains, with no clearly defined reduction zones. However, at ×500 magnification, small bright regions become distinguishable, and at ×1500 magnification, fine bright areas approximately 1–2 µm in size are clearly observed. These particles are distributed across the surface but retain a fine and dispersed structure, without forming a continuous metallic phase. This morphology indicates the initial growth of reduced iron, consistent with the partial reduction confirmed by X-ray diffraction analysis.

At 800 °C, a significant development of the metallic phase is observed. Even at ×50 magnification, the bright phase occupies most of the field of view; cracks have widened, and fractures have appeared. At higher magnification (×1500), large bright regions corresponding to aggregates of metallic iron grains are clearly visible. These grains begin to coalesce and form local metallic frameworks. The dark matrix, corresponding to silicate and phosphate phases, is still present but significantly reduced in volume. The morphology indicates active growth of the reduced iron phase and its emerging dominance within the material structure.

These observations are in good agreement with the results of the quantitative XRD analysis, which showed more than 90% metallic iron, and confirm that near-complete reduction of Fe is achieved at 800 °C. It is important to note that, as established in our previous work [1], phosphorus at this temperature remains in the oxide phase and does not transition into the metallic phase.

The sample reduced at 900 °C exhibits an almost continuous bright structure, with the exception of dark regions corresponding to quartz. The bright areas are larger and denser than those observed at 800 °C, indicating near-complete iron reduction. However, local EDS analysis presents challenges in accurately identifying the composition: Due to the high phase density and small scale, the electron beam simultaneously interacts with both metallic and oxide components. This results in background signal overlap (Al, Si, P), complicating interpretation. Despite these limitations, the visual features clearly indicate a high degree of reduction, although they do not allow for definitive conclusions regarding phosphorus behavior.

### 3.2. Magnetic Separation

In all experiments, significant mass losses were observed after magnetic separation (Table 1), primarily due to the fine non-magnetic fraction being carried away with distilled water. Because of the small particle size and their tendency to form stable suspensions, the non-magnetic portion did not settle completely and was difficult to filter—particularly at 800 and 900 °C. This significantly complicated the quantitative assessment of the non-magnetic fraction yield.

**Figure 8:**
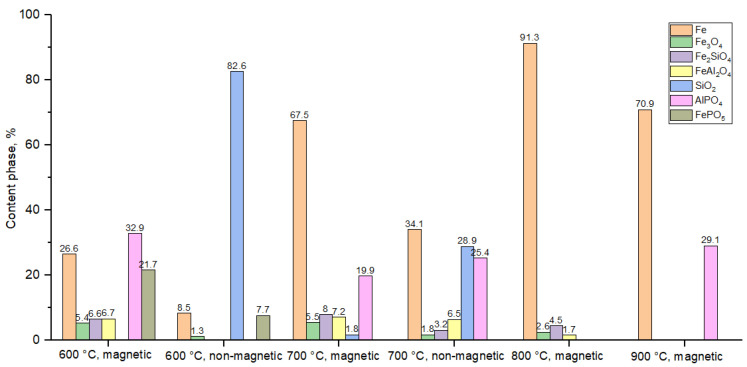
Phase composition after magnetic separation of hydrogen-reduced oolitic ore samples at 600, 700, 800, and 900 °C.

According to the XRD data (Figure 8), the magnetic fraction at all temperatures primarily contains metallic iron (Fe^0^), the proportion of which increases with the reduction temperature. However, in addition to metallic iron, phosphate phases (FePO_5_, AlPO_4_) were also detected in the magnetic fraction—especially at 600 and 900 °C. This indicates partial entrainment of phosphorus-containing phases during magnetic separation, likely in the form of inclusions or aggregates strongly bound to the iron phase.

The non-magnetic fraction obtained at 600 and 700 °C mainly consisted of oxide, silicate, and phosphate phases, including fayalite (Fe_2_SiO_4_), hercynite (FeAl_2_O_4_), FePO_5_, and AlPO_4_. Nevertheless, phosphates were also identified in the magnetic portion, indicating an inefficient distribution of phosphorus between the fractions.

Thus, magnetic separation does not provide selective separation of iron and phosphorus. The reasons include the following:

- inclusion of phosphates within iron grains;
- low magnetic contrast between the phases;
- aggregate bonding of phases after reduction;
- loss of the non-magnetic fraction, leading to underestimation of its content and composition.

The insufficient efficiency of magnetic separation for separating iron and phosphorus after hydrogen reduction suggests the need to replace or combine it with other beneficiation methods.

### 3.3. Liquid-Phase Separation

**Figure 9:**
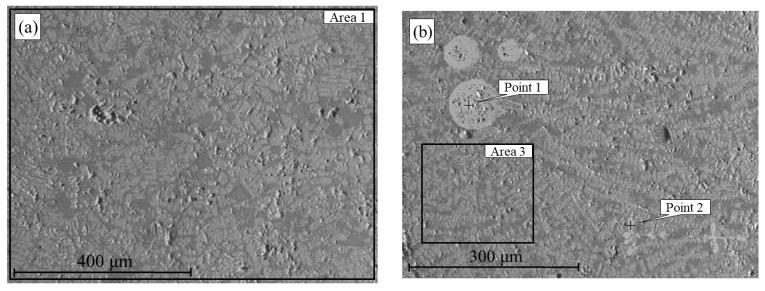
SEM images of samples obtained after smelting hydrogen-reduced oolitic ore at T, °C = 600 (**a**), 700 (**b**).

As part of the investigation into liquid-phase separation of hydrogen-reduced products, the microstructures and chemical compositions of the molten samples were analyzed. These samples were obtained by reducing the ore at 600, 700, 800, and 900 °C, followed by smelting at 1650 °C. Figure 9 presents SEM images of the solidified melts produced from materials reduced at 600 °C (a) and 700 °C (b).

The structure of the melt derived from the sample reduced at 600 °C appears visually heterogeneous, with signs of porosity and poorly defined phase boundaries. Clear separation into metallic and slag phases is not observed. According to the EDS analysis of region 1 (Table 2), the material contains 64.2 wt.% iron and 23.8% oxygen, as well as phosphorus, aluminum, and silicon. The presence of oxygen and other elements in significant concentrations indicates the persistence of oxidized phases and an insufficient degree of iron reduction at this temperature.

**Figure 10:**
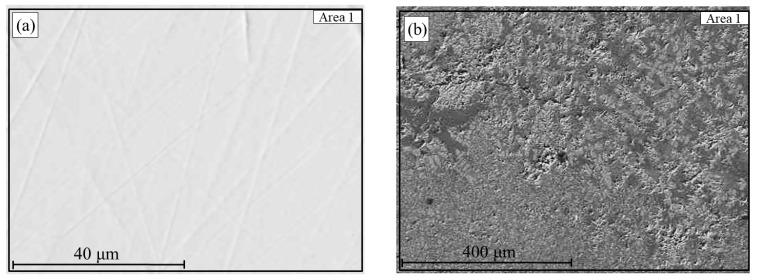
Metal (**a**) and slag (**b**) obtained after smelting of hydrogen-reduced oolitic ore samples at 800 °C.

Reduction at 800 °C provided favorable conditions for partial phase separation after smelting, as shown in the SEM images (Figure 10). The smelting process resulted in the formation of a metallic ingot and a separate slag phase. The metallic phase (Figure 10a) exhibits a dense, homogeneous structure and, according to EDS analysis (Table 3), contains 98.8 wt.% Fe and 1.2 wt.% P, indicating a high degree of iron reduction.

At the same time, in the slag phase (Figure 10b), local inclusions with high iron content are visually observed, likely representing fine metallic particles that did not coalesce with the main metallic ingot. This is supported by the EDS results: in the analyzed region 2, the composition includes 60.1 wt.% Fe and 20.1 wt.% O, along with Si, Al, and P. This distribution may be attributed to insufficient melt mobility under the given conditions or high viscosity of the slag phase, which hinders agglomeration and settling of the reduced iron into the metallic phase.

**Figure 11:**
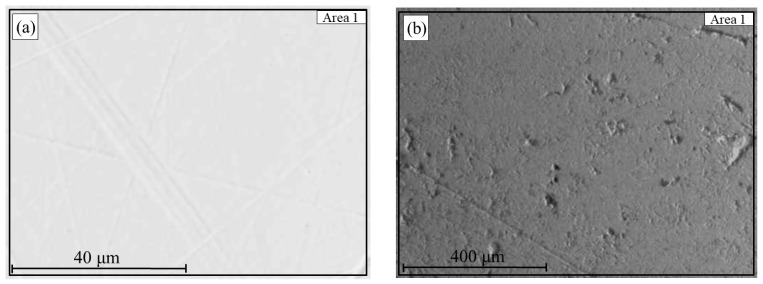
Metal (**a**) and slag (**b**) after smelting of hydrogen-reduced oolitic ore samples at 900 °C.

For the sample reduced at 900 °C, effective separation into metal and slag was also observed, with the mass of the metallic phase significantly higher than that at 800 °C (Figure 11). The SEM image of the metallic phase (Figure 11a) shows a dense, compact morphology typical of an alloy. According to EDS analysis (Table 4), the iron content is 98.5 wt.%, and phosphorus accounts for 1.5 wt.%. This indicates a high degree of iron reduction but also confirms that a portion of phosphorus transfers into the metal during smelting.

The slag phase (Figure 11b) is characterized by a homogeneous structure with lower iron content (18.2 wt.%) and elevated levels of oxygen (41.6 wt.%), silicon, aluminum, and phosphorus. In contrast to the sample reduced at 800 °C, no distinct local accumulations of metallic inclusions are observed in the slag, indicating more complete coagulation of the reduced iron and its settling into the metallic phase at the elevated temperature. Thus, reduction at 900 °C provides not only a higher degree of metallization but also improved phase separation due to the physicochemical properties of the system during smelting.

Thus, the results of liquid-phase processing of hydrogen-reduced samples showed that, at reduction temperatures of 600 and 700 °C, phase separation does not occur, and smelting yields a solid homogeneous mass. At 800 and 900 °C, it is possible to separate the metallic and slag phases, with the higher temperature promoting a greater yield of the metallic component. However, despite the selectivity of hydrogen reduction—where iron is predominantly reduced to the metallic phase while phosphorus remains in the oxide form—the experiments demonstrated that, during subsequent smelting, a portion of the phosphorus transfers into the metallic phase. This suggests that phosphorus may be reduced from phosphate compounds by metallic iron during smelting, resulting in partial rephosphorization of the metal.

To verify this hypothesis, a control experiment was conducted by smelting an artificial mixture of reduced iron and raw ore. The results are presented in the next section.

### 3.4. Phosphorus Reduction by Metallic Iron During Smelting

**Figure 12:**
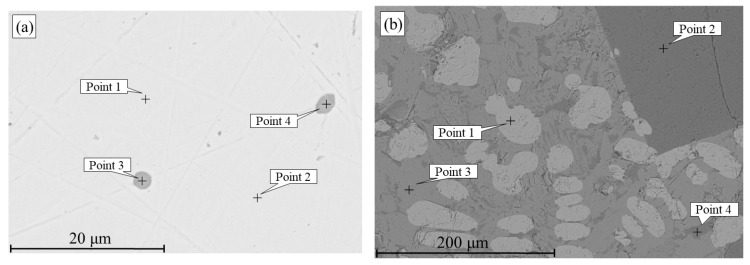
Metal (**a**) and slag (**b**) after smelting of a mixture of oolitic iron ore and reduced iron at 1650 °C.

Figure 12 and Table 5 shows the products obtained by smelting a mixture of Lisakovsk oolitic iron ore and reduced iron at 1650 °C. Micro-X-ray spectral analysis revealed that phosphorus was present in the metal as a solid solution in iron at concentrations of 0.4–0.5 wt.% (points 1 and 2, Figure 12a), as well as in spherical non-metallic inclusions. These phosphorus-containing inclusions consisted of 62.9–64.1 wt.% O, 10.7–11.5 wt.% P, and 24.4–26.3 wt.% Fe. The iron matrix and the non-metallic inclusions did not contain Al, Si, or Ca, indicating that other impurity elements remained in the slag phase.

The slag contains various phases differing in Fe, Al, Si, P, and Ca contents. For example, point 2 (Figure 12b) shows a relatively high aluminum content (20.2 wt.%), while point 3 (Figure 12b) has a relatively high silicon content (~11.9 wt.%). Point 1 (Figure 12b) is primarily composed of iron oxide. In point 4, 12.3 wt.% phosphorus and 9.3 wt.% calcium were detected.

**Figure 13:**
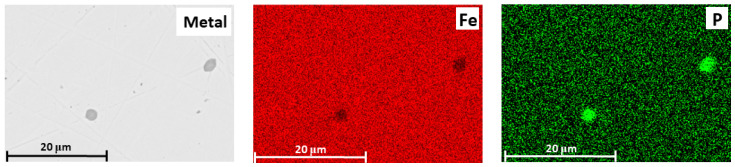
SEM image of the metal and elemental distribution maps (Fe and P) in the metal.

This pattern of phosphorus distribution among the phases is also confirmed by elemental distribution maps of the metal and slag. It is evident that during smelting of the mixture of high-phosphorus iron ore and reduced iron, phosphorus is uniformly distributed in the metallic phase and is also present in the non-metallic phase along with iron (Figure 13).

**Figure 14:**
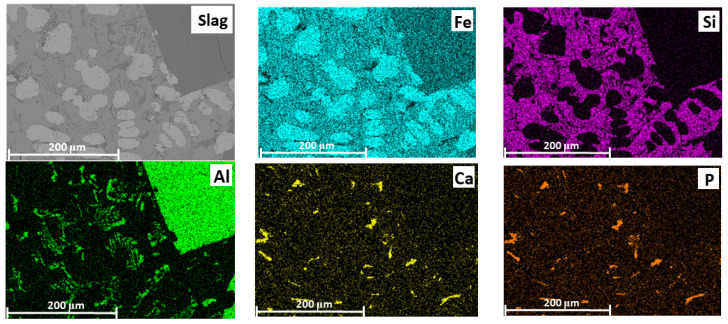
SEM image of the slag and elemental distribution maps (Fe, Si, Al, Ca and P) in the slag.

In the slag, iron is unevenly distributed across all phases (Figure 14). For example, in aluminum-rich phases, the iron content is relatively low compared to other areas. In regions containing silicon, the iron concentration is higher, possibly due to the formation of iron silicate compounds. Additionally, zones were identified that contained only iron and oxygen, with no significant amounts of aluminum or silicon—these areas likely correspond to iron in the form of FeO. Phosphorus is also unevenly distributed in the slag phase but is predominantly associated with calcium, indicating the formation of calcium phosphate compounds. To a lesser extent, phosphorus is associated with regions containing silicon and aluminum.

The obtained data confirmed the redistribution of phosphorus: as a result of smelting, part of the phosphorus migrated from the oxide phase into the metal, which supports the possibility of phosphorus reduction from its compounds by metallic iron.

The mechanism of phosphorus reduction by metallic iron, from the perspective of the electronic theory of reduction [32], can be explained by electron transfer from metallic iron to phosphorus cations. Considering that phosphorus is present in the slag in an oxidized state as the cation 2P^5+^, each phosphorus cation must regain five electrons to transition into the metallic state and form a metallic bond. This process corresponds to the reaction (2P^5+^) + 10e^−^ = 2[P^0^]. In the absence of other reducing agents capable of donating valence electrons to reducible cations, phosphorus cations can acquire the necessary electrons only from pre-reduced metallic iron. Consequently, phosphorus reduction is coupled with the oxidation of iron according to the reaction [Fe^0^] = Fe^2+^ + 2e^−^. Therefore, the overall redox reaction can be represented as 2(P^5+^) + 5[Fe^0^] = 2[P^0^] + 5(Fe^2+^), meaning that when the temperature is raised to the level required for melting the products of solid-state metallization, metallic iron reduces phosphorus.

Thus, the conducted liquid-phase separation experiments demonstrated that despite the effective reduction of iron, the selectivity of the process is compromised during the smelting stage, where phosphates undergo secondary reduction by metallic iron. This leads to the partial or complete incorporation of phosphorus into the alloy, making the liquid-phase stage unsuitable for separating reduced iron from phosphorus without the use of additional slag-forming agents or modification of smelting conditions.

## 4. Conclusions

This study investigated the processes of selective reduction of iron from high-phosphorus oolitic ore using hydrogen at temperatures ranging from 600 to 900 °C, followed by liquid-phase separation of the reduced products. It was established that at 600 and 700 °C, iron’s reduction is incomplete, and no distinct separation into metallic and slag phases is observed after smelting. In contrast, at 800 and 900 °C, more pronounced phase separation occurs, with the metallic-phase yield being higher at 900 °C.

However, even in cases of visually distinct separation between metal and slag, the metallic phase retains a significant phosphorus content (up to 1.5 wt.%), indicating that phosphorus is reduced during the smelting stage. In the slag, phosphate compounds were observed, primarily associated with calcium, aluminum, and silicon, while at 800 °C, individual inclusions of reduced iron that did not coalesce into the main metallic phase were detected.

A control experiment involving the smelting of a mixture of reduced iron and raw ore confirmed the possibility of phosphorus reduction by metallic iron at 1650 °C. Based on the electronic theory of reduction, this process can be explained by electron transfer from Fe^0^ to phosphorus in phosphate groups (P^5+^), leading to phosphorus’s reduction to its elemental state and subsequent incorporation into the alloy.

Thus, despite the effectiveness of selective hydrogen-based iron reduction, subsequent liquid-phase separation without phosphorus stabilization in the slag does not ensure adequate phosphorus removal. To enhance selectivity, smelting conditions must be improved to prevent phosphorus’s reduction by iron—such as through slag composition modification or the introduction of fluxing agents that bind phosphorus into stable compounds.

## Figures and Tables

**Table 1:** Mass balance of fractions after wet magnetic separation.

Temperature, (°C)	Mass of Magnetic Fraction, (g)	Mass of Non-Magnetic Fraction, (g)	Total Mass, (g)	Mass of Losses, (g)	Mass of Losses, (%)
600	12.55	3.71	16.26	3.74	18.70
700	10.88	4.64	15.52	4.48	22.40
800	14.51	–	14.52	5.49	27.45
900	14.98	–	14.98	5.02	25.10

**Table 2:** Elemental composition (wt.%) of the molten product obtained after hydrogen reduction at 600–700 °C followed by smelting.

Sample	Designation	O	Al	Si	P	Ca	Fe
600 °C (Figure 9a)	Section 1	23.8	5.4	5.0	1.2	0.4	64.2
700 °C (Figure 9b)	Point 1	–	–	–	–	–	100
700 °C (Figure 9b)	Point 2	–	–	–	–	–	100
700 °C (Figure 9b)	Section 3	25.8	6.9	5.9	1.4	0.4	59.6

**Table 3:** Elemental composition (wt.%) of the molten product (a—metal, b—slag) obtained after hydrogen reduction at 800 °C followed by smelting.

Sample (Figure 10)	Designation	O	Mg	Al	Si	P	Ca	Cr	Mn	Fe
800 °C (a)	Section 1	–	–	–	–	1.2	–	–	–	98.8
800 °C (b)	Section 1	20.1	1.2	9.7	6.0	1.6	0.5	0.3	0.5	60.1

**Table 4:** Elemental composition (wt.%) of the molten product (a—metal, b—slag) obtained after hydrogen reduction at 900 °C followed by smelting.

Sample (Figure 11)	Designation	O	Mg	Al	Si	P	Ca	Cr	Mn	Fe
900 °C (a)	Section 1	–	–	–	–	1.5	–	–	98.5	–
900 °C (b)	Section 1	41.6	1.2	13.8	19.2	4.1	2.1	0.4	18.2	41.6

**Table 5:** Elemental composition of metal and slag (wt.%).

Designation	O	Al	Si	P	Ca	Fe
Point 1 (Figure 12a)	-	-	-	0.4	-	99.6
Point 2 (Figure 12a)	-	-	-	0.5	-	99.5
Point 3 (Figure 12a)	62.9	-	-	10.7	-	26.3
Point 4 (Figure 12a)	64.1	-	-	11.5	-	24.4
Point 1 (Figure 12b)	58.6	-	-	-	-	41.4
Point 2 (Figure 12b)	63.5	20.2	-	-	-	16.3
Point 3 (Figure 12b)	64.1	-	11.9	-	-	24.0
Point 4 (Figure 12b)	68.7	-	-	12.3	9.3	9.8

## Data Availability

The original contributions presented in this study are included in the article. Further inquiries can be directed to the corresponding authors.
